# Critical care and the law: Pertinent cases from 2023

**DOI:** 10.1177/17511437251346304

**Published:** 2025-06-17

**Authors:** Aaron D’Sa, Robert Tobin, Luigi Camporota, Thearina de Beer, Dan Harvey, Richard Innes, Prashanth Nandhabalan, Victoria Metaxa

**Affiliations:** 1 University of Cambridge School of Clinical Medicine, Cambridge, UK; 2 Nottingham University Hospitals NHS Trust, Nottingham, UK; 3 King’s College Hospital NHS Foundation Trust, London, UK; 4 Nottingham Universities NHS Trust, Nottingham, UK; 5 Kennedys Law LLP, London, England, UK; 6 Royal Free Hospital, London, UK; 7 Somerset NHS Foundation Trust, Taunton, England, UK; 8 Guy’s and St Thomas’ Hospitals NHS Trust, London, England, UK

**Keywords:** Intensive care, law and ethics, coroner, human rights

## Abstract

This review by the Legal and Ethical Advisory Group (LEAG) summarizes the important legal cases and prevention of future deaths (PFDs) ruled or issued in 2023 that are pertinent to Intensive Care Medicine. The legal cases include human rights cases, clinical negligence and rulings of the court of protection. Not all of the cases relate to events which have occurred in intensive care, however the rulings will have a bearing on intensive care practice.

## Introduction

Broadly there are four types of legal cases and reports relevant to the Intensive Care community included in this summary. The first are cases relating to *human rights*. These cases cut across traditional legal boundaries, and pertain to subjects such as consent, capacity, autonomy and privacy. The second type relates to the *law of negligence*, that is the legal mechanism by which patients are compensated for harm caused by a breach of a practitioner’s or organization’s duty of care. The third type relates to the *court of protection*, which is responsible for a final decision on whether a patient has capacity and for determining a patient’s best interests, in the event of a disagreement. Finally, coroner’s *prevention-of future-deaths* reports are useful to highlight issues that have arisen in the coroner’s court which may relate to Intensive Care practice.

This paper reviews the legal rulings in 2023, which are of relevance to the Intensive Care community. The summaries focus on the aspects of the cases relevant to practicing intensivists, referring to court procedures only where such details would be of material assistance. To aid the reader’s understanding of the application of the law we have contextualized the summaries with relevant legislation or case law.

## Human rights cases

The European Convention on Human Rights, of which the UK is a signatory, defines a set of rights or entitlements conferred to everyone and which are protected against violation by the state. The full set of the Convention Articles, which outline the full range of rights protected, can be found in the references.

Articles 8 and 10 protect the right to privacy and the right to freedom of expression, respectively. There is an inherent tension between these rights, and this tension has become evident in Intensive Care as patients and their families have sought to publicize their experiences and draw attention to the acts of particular, named intensive care clinicians.

*Abbasi v Newcastle* and *Haastrup v KCL*, were two related cases pertaining to the withdrawal of life-sustaining treatment for children admitted to Intensive Care Units (ICUs). The court issued a reporting restriction order (RRO) to prevent disclosure of the names of the healthcare professionals involved in the cases. The original orders were of indefinite duration. These orders were challenged first in the High Court (unsuccessfully) and again (successfully) in the Court of Appeal, by the families of the children, who wanted to be able to tell their story.

Lord Chief Justice Burnett, in the Court of Appeal, explained the purpose of the RROs:

‘Reporting Restriction Orders often protect the identities of all those involved in the care of a patient in respect of whom an application to withdraw treatment is made. That is usually to protect the privacy of the patient, of the patient’s immediate family and of those concerned in the treatment of the patient as well as to safeguard the integrity of the proceedings. Such proceedings are apt to generate a great deal of passionate debate which spills over into harassment of those involved in the proceedings, picketing of hospitals and interference with the working of the hospitals’. – para 1

The Court of Appeal set aside the RRO. In doing so the court balanced the clinicians’ Article 8 right to privacy, and the family’s article 10 right to freedom of expression. The original legal cases (pertaining to the withdrawal of life-sustaining treatment) had long finished, and it was thought that there was limited risk posed to the clinicians’ right to privacy by being publicly identified. Lord Burnett drew attention to the protections available to those clinicians who might be identified:

‘The civil and criminal law both provide protection from various aspects of online attack, some preventative and other to provide a remedy for legal wrongs. To that extent nobody is obliged simply to ‘put up with’ abuse. However, the courts cannot shut down legitimate debate save when the rights of those affected by that debate, or put differently the adverse consequences, are of such strength as to outweigh the right to free expression’. – para 121

In support of setting aside the RRO, the court referenced a strong public interest in discussion of the cases, both of which related to the morality and ethics of decision-making for vulnerable people at the end of their lives.

*University Hospitals Birmingham v Thirumalesh*, also concerned the tension between the right to privacy for clinical staff, and the right to freedom of expression for families. The Court of Protection had ruled (in *NHS Trust v ST*, a case discussed below) that a 19-year-old patient lacked capacity. As part of the ruling, a Transparency Order was issued which

‘*prohibits the publication (by any means, direct or indirect) of the names of, or any information that would identify, ST, the family of ST, the applicant Hospital Trust, the hospital(s) attended by ST, experts, treating clinicians and any health/care professional engaged with ST*’. – para 4

This order was challenged by the patient’s family in *University Hospitals Birmingham v Thirumalesh*, who wanted to draw attention to defects in the patient’s care. The Court accepted the relaxation of the Transparency order to allow identification of the patient and her family, but was more restrictive in relaxation of the order in relation to the identification of staff. Mr Justice Peel explained

‘The publicity generated by this case has been heated in some quarters. There is likely to be heightened interest in the coming days as a result of my intention that the restrictions on identifying ST and her family should be immediately lifted. If anonymisation of clinicians is lifted, the consequences are unpredictable, but there is in my judgment a risk that abuse and harassment may follow, particularly as they may be reported by the family as having given ST inadequate care. Were that to come to pass, I would regard it as a very considerable interference with their Article *8* rights. That risk is likely to be at its most acute in the next few weeks and I consider that there should be a “cooling off period” measured in weeks. That would be a proportionate interference with the family’s and the media’s Article 10 rights, given the potential interference with the clinical/nursing staff Article *8* rights’. – para 43

The three cases were the subject of an appeal to the Supreme Court. The judgement ([2025] UKSC 15) confirmed that ICU staff do not have an absolute right to privacy, and the risk of infringement of that right will be considered case-by-case. Clinicians working in public hospitals are, for the purpose of the Convention, public figures and the treatment of patients in public hospitals is a matter of legitimate public interest and debate.

The protection of clinicians from unfounded accusations and abuse is also a legitimate aim, and when balancing the Article 8 rights (of clinicians) and Article 10 rights (of the patient’s family) the potential for attacks on staff and the impact of such attacks on morale and recruitment, are relevant. Stronger protections may be afforded while a case is ongoing than when media interest has abated. The protections afforded by the civil and criminal law, as well as by the police, will be considered by the court in deciding whether the anonymity of clinical staff requires protection.

The Supreme Court judgement also confirmed that the rights of the clinicians are not subsidiary to the rights and interests of the child, but are deserving of direct protection. It follows that applications to protect these rights should be brought by the clinicians themselves, although the employing trust may be best placed to initiate proceedings on the clinicians’ behalf. In the latter case, the clinicians should be joined as parties early in the proceedings so that they have the opportunity to assert their rights themselves.

## Negligence cases

The law requires all clinicians to take reasonable care in the discharge of their professional duties. What is meant by reasonable care will of course vary according to circumstances, but the standard for most professional activities is set by the Bolam test. This familiar test, outlined by McNair J in Bolam v Friern, holds that a responsible practitioner whose practice accords with that of other professionals ‘skilled in that particular art’, is not negligent. Two notable consequences of this test are that, firstly, a practice is not inadequate purely by virtue of being a minority approach, and secondly that the standard is set by professionals themselves. The Montgomery case highlighted that some professional activities, namely the disclosure of the risk of different treatment options, are not purely based on professional skill and so should not be judged according to the Bolam test.

*McCulloch and others (Appellants) v Forth Valley Health Board* clarified that deciding which treatment options might be appropriate to offer to a patient is purely an exercise in professional skill. It follows that the Bolam test is appropriate for determining the standard of care.

The difference between Montgomery and McCulloch in a critical care context can be illustrated as follows: where a patient is admitted to ICU with renal impairment, many treatment strategies might theoretically be helpful to manage the renal impairment. Deciding which of these options should be offered to the patient is an exercise in professional skill, and the way in which a clinician selects or rejects therapies to formulate the final ‘menu’ will – if criticized – be judged according to the Bolam test.

Once the patient is presented with the options, the disclosure of the risks associated with each one is not purely an exercise of professional skill, but must also consider the patient’s own preferences, interests and concerns. These should be elicited directly from the patient, and not assumed. This reflects GMC guidance on the disclosure of information during joint decision-making, which can be found here. The disclosure of risk during the consent process is then judged according to the more sophisticated Montgomery test.

## Capacity and best interests

The Intensive Care clinicians have considerable experience caring for patients who lack capacity, and whose healthcare decisions are governed by the Mental Capacity Act 2005. Unsurprisingly, a significant number of Court of Protection cases relate to patients who are either admitted to or are being considered for treatments ordinarily provided in ICU.

*Hemachandran v Thirumalesh* concerns the withdrawal of life-sustaining treatment of a 19-year-old patient. The reporting restrictions element of this case has already been discussed above. The Court of Appeal ruling relating to the functional test for capacity was handed down in 2024, however the High Court ruling which was appealed was handed down in 2023 and on that basis the case is considered here.

The patient had been born with a mitochondrial disorder, which had resulted in a neurodegenerative disease with no known cure. The patient did not believe she was dying and wished to pursue experimental nucleoside therapy, which was not available at the Birmingham Trust, where she had been admitted to ICU. The patient’s treating clinicians felt that the patient did not have capacity and sought a declaration from the Court of Protection to this effect, which was granted.

Unfortunately, the patient died soon afterwards, however permission was granted for the patient’s family to challenge the declaration of incapacity, as the challenge raised important issues around the nature of the statutory capacity test.

The court divided the familiar capacity test contained within the MCA into a *diagnostic or impairment test* (hereafter the impairment test), and a *functional test*. The impairment test, contained within Section 2 of the MCA, requires that the person is unable to make a decision because of an impairment of their mind or brain.

Section 3 of the MCA describes the functional test, specifying that a person is unable to make a decision if they cannot understand, retain or weigh relevant information or communicate their decision. The consensus of medical opinion was that the patient was dying. The trust argued that this was a relevant fact and claimed that since the patient did not believe it, they could not weigh it. The patient would therefore meet the criteria of the functional test and consequently lacked capacity.

Lady Justice King disagreed, preferring the approach put forward by the official solicitor:

‘a person who does not believe relevant information, whether it be factual or opinion, may lack capacity, but equally they may not. The meaning of each of the words ‘understand’, ‘use’ and ‘weigh’ is, she submits, different from the meaning of the word ‘believe’. – para 58

The ruling clarifies that a patient’s disbelief of pertinent facts may be relevant to, but is not determinative of, whether they can understand or weigh relevant information for the purposes of making a decision.

The ruling in *Kings College Hospital NHS Foundation Trust v X and Y* is helpful in clarifying that a patient’s wishes – in so far as they can be determined – are not determinative of their best interests. The patient had unfortunately suffered a catastrophic brain injury from a road traffic collision. The Trust argued that, as the patient was in a persistent vegetative state with no prospect of recovery or ability to perceive anything, withdrawal of life-sustaining treatment was in the patient’s best interests.

The patient’s family argued that the patient would not have wanted to ‘give up’, providing evidence of the patient being brought up in the Christian faith, which requires that people die naturally rather than by withdrawal of treatment. The court accepted that the patient had a ‘determined and resilient nature’ and that the court could infer that he would have wanted withdrawal of treatment to be delayed. Notwithstanding this inference, a withdrawal of treatment was held to be in the patient’s best interests, with the patient’s imputed wishes not being determinative.

*The NHS Foundation Trust v K* is brought to the attention of the Intensive Care community for reasons highlighted by a useful case note by 39 Essex Chambers, namely that it highlights how a Trust’s clinicians may helpfully outline the range of options available to assist the court’s decision-making.

The patient in this case was admitted to ICU for a progressive condition and the opinion of her treating clinicians was that she was approaching the end of her life. The treating clinicians sought a declaration of best interests, outlining three treatment options which they felt might potentially be applicable. In summary, these were continued mechanical ventilation via an oral endotracheal tube, extubation and palliation, or a tracheostomy. The trust clinicians offered to support the second and third option, and argued that the second was in the patient’s best interests. As the case note points out, the circumstances of the case are not unusual in ICU however the trust’s approach in outlining the theoretical, supportable, and preferred options was helpful to the court and would be a useful approach in future cases.

## Prevention of future death (PFD) reports

Strictly these are not court rulings, as they arise from a statutory duty to issue reports where issues highlighted in a coronial inquest have the potential to cause further loss of life if not corrected. The reports are issued under Regulation 28 of The Coroners (Investigations) Regulations 2013, and so are sometimes referred to as Regulation 28 Reports.

### Ref: 2023-0519 Deceased name: Ian Jacka

This PFD highlighted an inadequate handover of the critical care team to the surgical and anaesthetic team prior to spinal surgery. The handover omitted details of a life-threatening critical incident that had occurred the day before surgery, which increased the patient’s vulnerability to anaesthetic and surgical interventions. Both intensive care admissions and surgical procedures represent periods of significant risk. The complexity and duration of intensive care admissions make it difficult to provide a summary that is both manageable and comprehensive. An exceptional handover for one team may be inadequate for another.

### Ref: 2023-0489 Deceased name: Katherine Flynn

This case is of interest to neuro-ICUs, highlighting the absence of national policy for the management of external ventricular drains. In this case the trust’s guidance was not clear on the escalation process for a drain that had stopped draining but was still oscillating.

### Ref: 2023-0324 Deceased name: Cherry Garland

This case describes the patchwork implementation of digital patient notes across different trusts, and within different departments in the same trust.

In this case, the ICU utilized an electronic prescribing system which was not used elsewhere in the hospital. This necessitated transcription of the ICU prescriptions manually onto a paper chart each time a patient was discharged from ICU. Though the transcription error in this case (a failure to correctly continue antibiotics) was not thought to have a material impact on the patient’s care, the system (of manual transcriptions) as revealed to the coroner was thought to present such a risk to other patients as to warrant a PFD report.

### Ref: 2023-0251 Deceased name: Jane Wadsworth

This case concerns the difficulty experienced by hospital departments in making an ICU referral on bank holidays. The ward team had found it difficult to obtain a doctor-to-doctor discussion regarding a critical care referral. The case’s complexity meant that support on the ward via the critical care outreach team was not thought appropriate, and staff shortages meant the option was not available in practice. Staff shortages are a national issue that is certainly not specific to ICU, however the case highlights that the critical nature of ICU services means that intensivists and outreach teams should be adequately resourced and supported.

### Ref: 2023-0219 Deceased name: Matthew Phipps

The coroner in this case identified that the ICU caring for the patient did not have an adequate escalation policy to ensure that patients that require escalation of treatment can receive it, even when the ICU is fully occupied. The report does not expect the impossible, highlighting that some of the patients in the ICU were fit enough to be discharged and might have been moved to alternative areas to improve capacity.

## Conclusions

In this article we present a list and summary of pertinent legal cases ruled by the courts in 2023 which, although not exhaustive, is hopefully informative to the critical care community. It provides insight into the obligations of clinicians when offering treatment options to patients and the extent to which their professional skills can set standards; it describes the proceedings in cases of disagreement with families when the patient lacks capacity and the weight of personal wishes when limitation of treatment is considered; it cautions about the difficult balance between the right to privacy and the right to freedom of expression in cases with heightened media interest. Knowledge of the legal landscape will hopefully complement the medical expertise of ICU clinicians and allow them to practice safely and confidently.

**Legislation and Conventions**

European Convention on Human Rights (coe.int)

Human Rights Act 1998

**Guidance**

Decision Making and Consent, General Medical Council (2024)

**Case Notes**

Mental Capacity Report February 2024 HWDOL.pdf (39essex.com)

**Case Law**

Abbasi & Anor v Newcastle Upon Tyne Hospitals NHS Foundation Trust [2023] EWCA Civ 331 (31 March 2023) (bailii.org)

Abbasi and another (Respondents) v Newcastle upon Tyne Hospitals NHS Foundation Trust (Appellant); Haastrup (Respondent) v King’s College Hospital NHS Foundation Trust (Appellant) [2025] UKSC 15

Bolam v Friern Hospital Management Committee (1957) 1 WLR 583

Hemachandran final judgement CA 2023 001892 (judiciary.uk)

Kings College Hospital NHS Foundation Trust v X & Anor [2023] EWCOP 34 (04 August 2023) (bailii.org)

McCulloch and others (Appellants) v Forth Valley Health Board (Respondent) (Scotland) (supremecourt.uk)

Montgomery v Lanarkshire Health Board [2015] UKSC 11

The NHS Foundation Trust v K & Ors [2023] EWCOP 57 (14 December 2023) (bailii.org)

ST -v- University Hospitals Birmingham judgment (judiciary.uk)

